# Impurity Resonant States p-type Doping in Wide-Band-Gap Nitrides

**DOI:** 10.1038/srep19537

**Published:** 2016-01-18

**Authors:** Zhiqiang Liu, Xiaoyan Yi, Zhiguo Yu, Guodong Yuan, Yang Liu, Junxi Wang, Jinmin Li, Na Lu, Ian Ferguson, Yong Zhang

**Affiliations:** 1 Research and Development Center for Solid State Lighting, Institute of Semiconductors, Chinese Academy of Science, Beijing, 100086, China; 2 State Key Laboratory of Solid State Lighting, Beijing, 100086, China; 3 School of Physics and Engineering, Sun Yat-Sen University, Guangzhou 510275, China; 4 Lyles School of Civil Engineering, Purdue University, West Lafayette, IN 47907, USA; 5 College of Engineering and Computing, Missouri University of Science and Technology, 305 McNutt Hall, 1400 N. Bishop, Rolla, MO 65409, USA; 6 Department of Electrical and Computer Engineering, The University of North Carolina at Charlotte, 9201 University City Blvd., Charlotte, North Carolina 28223, USA

## Abstract

In this work, a new strategy for achieving efficient p-type doping in high bandgap nitride semiconductors to overcome the fundamental issue of high activation energy has been proposed and investigated theoretically, and demonstrated experimentally. Specifically, in an Al_x_Ga_1−x_N/GaN superlattice structure, by modulation doping of Mg in the Al_x_Ga_1−x_N barriers, high concentration of holes are generated throughout the material. A hole concentration as high as 1.1 × 10^18^ cm^−3^ has been achieved, which is about one order of magnitude higher than that typically achievable by direct doping GaN. Results from first-principle calculations indicate that the coupling and hybridization between Mg 2*p* impurity and the host N 2*p* orbitals are main reasons for the generation of resonant states in the GaN wells, which further results in the high hole concentration. We expect this approach to be equally applicable for other high bandgap materials where efficient p-type doing is difficult. Furthermore, a two-carrier-species Hall-effect model is proposed to delineate and discriminate the characteristics of the bulk and 2D hole, which usually coexist in superlattice-like doping systems. The model reported here can also be used to explain the abnormal freeze-in effect observed in many previous reports.

Group III-nitride semiconductors possess a number of excellent properties including a tunable, direct band gap, high drift velocity, high mobility, and strong light absorption^1^,^2^,^3^,^4^. Such properties make them viable for a broad range of electronic and optoelectronic devices and applications. Despite the tremendous progress which has been made in the growth and fabrication of such Group III semiconductors, achieving a high p-type conductivity in nitrides has been shown to be extremely difficult, which hinders further improvement in the performance of nitride-based devices. It is well known that, similar to most wide-band-gap semiconductors such as diamond and ZnO, nitrides have a “unipolar” or “asymmetric” doping problem. This can be attributed to low dopant solubility, hydrogen passivation, relatively low valence-band maximum (VBM) and high defect ionization energies^5^,^6^,^7^. Considerable effort has been expended to address this p-type doping issue in Group III-nitrides^8^,^9^,^10^. Recent advances in crystal growth technology have shown that the issues of low solubility and hydrogen passivation can, at least to some extent, be overcome by using non-equilibrium growth techniques and high-temperature annealing. However, alleviating the more fundamental problem of high activation energies has, to date, not yet been satisfactorily achieved. The underlying physical mechanism in this problem is attributed to the electronic structure of the host material. Nitrogen is strongly electronegative and has a deep 2p atomic orbital. Thus, the valance band maximum (VBM) of nitrides, which contain mostly N 2p orbitals, is at relatively low energies. This leads to a relatively deep acceptor energy level which makes it very inefficient for thermal activation. To date, the most promising acceptor for III-nitrides continues to be Mg. Unfortunately, even with Mg dopant ions, the activation energy *E*_a_ of the Mg dopant in GaN is still in the range of 160 and 200 meV. For AlN, the activation energy can be as high as 630 meV. Consequently, only a small fraction of Mg can be activated at room temperature^11^,^12^.

Various approaches have been sought to lower the acceptor levels and reduce the acceptor ionization energy in nitrides. Recently, B. Gunning *et al.* proposed a strategy for lowering the acceptor impurity states by extremely high doping^4^. They argue that, as the electrically active acceptor concentration increases, the isolated deep acceptor levels begin to interact and split into an impurity band, which is closer to the valence band thus lowering the effective activation energy. Peter and Schubert^13^,^14^ demonstrated another strategy and found that by polarization induced modulation of the valence band edge in a superlattice, the low doping efficiency could be partially overcome. Simon and Jena^15^ also suggested that a 3D hole gas could be produced using the built-in electronic polarization in nitrides. However, in these previous works more direct evidence is required to further delineate and discriminate the characteristics of the 3D and 2D hole gases, which usually coexist in superlattice-like doping systems, for instance multiple–quantum-well structures, compositionally graded layer structures, or heterojunction interfaces^13^,^14^,^15^,^16^,^17^. Elevating the VBM of the host material by co-doping has been regarded as another strategy to address this issue^8^,^18^, for example by Si-Mg co-doping and mutually passivated defect pair co-doping. However, intensive theoretical analyses show that this type of energy level coupling is too small to significantly reduce the acceptor ionization energy due to different symmetries and wave-function characteristics^10^. Therefore, although partial successes have been achieved, the mechanisms of those methods are still controversial and poorly understood. Better approaches or alternative strategies to create more stable and shallower acceptors in nitrides are highly desired.

As discussed above, the behavior of Mg as an acceptor in nitride semiconductors is strongly linked to the position of the Mg impurity states related to the VBM of the host materials. Besides co-doping, a periodic oscillation of the valance band edge produced by a superlattice structure, such as Al_x_Ga_1−x_N/GaN, can also modify the characteristics and energy position of the VBM^13^,^14^. Based on this consideration, a novel strategy for efficient p-type doping is proposed to overcome the fundamental problem of high activation energy by inducing impurity resonant states in an Mg doped Al_x_Ga_1−x_N/GaN superlattice structure. As schematically shown in Fig. 1, in the structure developed using our proposed strategy, the discrete wave-functions of Mg impurity states are able to overlap to form continuous miniband-like impurity states^19^,^20^. Therefore, the initially localized impurity states in Al_x_Ga_1−x_N barrier layers form resonant states in the GaN layer (i.e. with energy levels below or close to the GaN VBM, it strongly depends on the Al percentage in Al_x_Ga_1−x_N). To see the exact energy position of Mg impurity state, one would need to use a pretty large cell. Alternatively, in this work we offer the above qualitative band-diagram to explain the idea of resonant state p-type doping. In the case of considerable acceptor density, these impurity states are broadened^4^,^21^,^22^,^23^, which can further enhance the coupling between them. In this new scenario, electrons are able to drop from the VBM of GaN into the impurity states or band without any energy barrier, which means the acceptors are self-ionized. Hence, high concentration of the acceptors can be expected. In addition, as proposed by previous reports, the polarization effect also enhances the ionization of the deep acceptors and leads to the accumulation of carriers as a hole sheet, which further increase the effective hole concentration in the host materials^13^,^14^,^15^. In this work, to test these proposed concepts, the impact of impurity resonant states on the ionization energy of Mg acceptors is analyzed through both theoretical and experimental methods.

## Results

### The characteristics of Mg impurity resonant states

**Figure 2:**
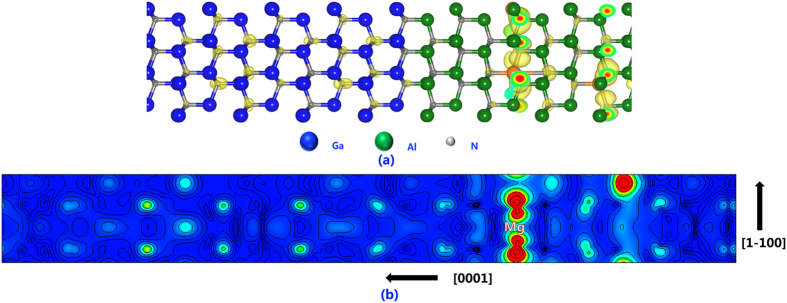
Distribution of Mg impurity states. Isosurface charge density plots of Mg impurity states at Γ point in Al_x_Ga_1−x_N/GaN. (**a**) atomic configuration and isosurface charge density of Mg impurity states, (**b**) isosurface charge density of Mg impurity states in m plane.

**Figure 3:**
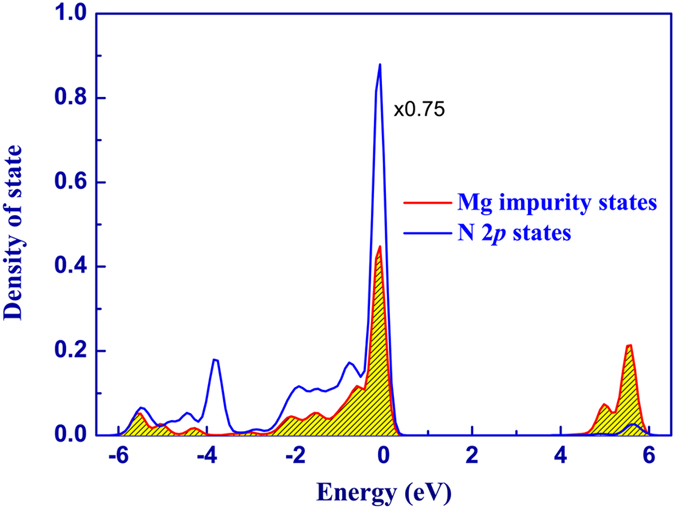
Evidence for the delocalization characteristics of Mg impurity states. Calculated projected density of states of Mg 2*p* impurity states and N 2*p* states.

To understand the characteristics and distribution of Mg impurity states, the charge density of the Mg impurity states at the Γ point are plotted in Fig. 2. As can be expected, most of the charge density is accumulated around the Mg atoms. However, it cannot be ignored that a significant amount of Mg impurity states become delocalized and distributed in both barrier and well. In term of the well, such Mg impurity orbitals lie inside the valance band and act as resonant states. Now, the only question left to consider is whether the discrete impurity states in different barrier layers can couple with each other. In a previous report, E.F. Schubert assumed that the acceptor-effective Bohr radius is much smaller than the period of the superlattice, and argued that the accepter levels in the barriers are not influenced by the adjacent barriers^14^. In fact, the acceptor Bohr radius is not directly relevant to the Mg impurity level and its coupling with the host^24^. In the nitride matrix environment, the Mg impurity states and host N 2*p* states can couple strongly with each other since they each share the same t_*2p*_ symmetry, and hybrid orbitals are formed. As a result, now the Mg impurity states will also contain the characteristics of N 2*p* orbitals and become delocalized to some extent. The distribution of Mg impurity states in both barrier and well is direct evidence to support our theory proposed above. To understand the mechanism of orbital hybridization between Mg and N, projected densities of states (DOSs) were analyzed and are shown in Fig. 3. As can be seen, several peaks of Mg 2*p* states, especially near the VBM overlap with that of N 2*p* are observed, which indicates the coupling between them^25^,^26^. Therefore, we suggest that hybridization of the Mg and N 2*p* states should be the reason for the occurrence of impurity states lying in the well.

### Preparation and characterization of Al_x_Ga_1−x_N/GaN superlattice structures

**Figure 4:**
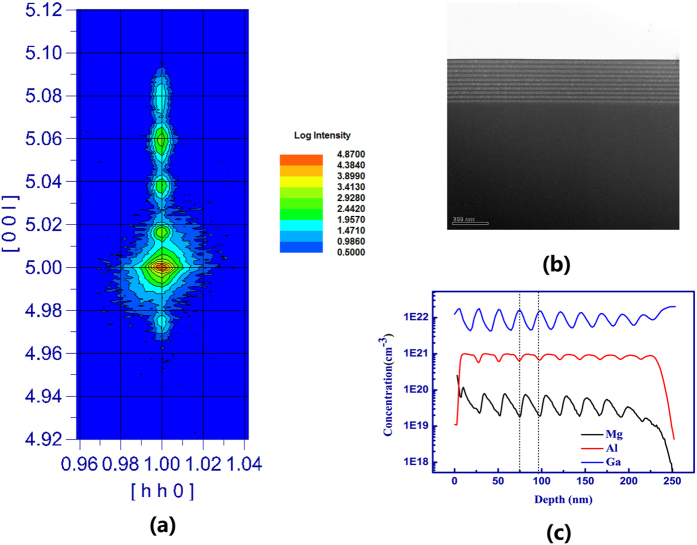
Structure and crystalline quality of Al_x_Ga_1−x_N/GaN sample. (**a**) asymmetrical (105) X-ray reciprocal space mapping, (**b**) TEM image of our AlxGa_1−x_N/GaN sample, (**c**) SIMS depth profiles of Mg for AlxGa_1−x_N/GaN sample.

To further test the concept of impurity resonant state p-type doping, Al_x_Ga_1−x_N/GaN superlattice structures were grown by metal-organic chemical vapor deposition (MOCVD) on a c-plane sapphire substrate. After depositing a low-temperature GaN nucleation layer on the sapphire substrate, a 3 μm undoped GaN layer was grown. Then, the Al_x_Ga_1−x_N/GaN superlattice was deposited upon the undoped GaN layer. The barrier and well thickness were both 10 nm with total 10 periods. To avoid the conventional thermal ionization mechanism of Mg dopant in GaN layers, only Al_x_Ga_1−x_N was Mg doped. The aluminum percentage in the barrier was fixed at 30%, a value typically used in GaN LED structures. The sample structures were characterized by TEM and asymmetrical (105) X-ray reciprocal space mapping (RSM). As shown in Fig. 4(a), the main GaN peak and the zero-order diffraction satellite peak of the Al_x_Ga_1−x_N /GaN MQWs are aligned in a vertical line parallel to the Q_y_ axis, indicating the 30% AlGaN films is almost completely strained without relaxation along the plane direction. The high crystalline quality of our sample can also be confirmed by TEM image shown in Fig. 4(b).

Furthermore, as shown in Fig. 4(c), secondary ion mass spectrometry measurements were performed to verify the incorporation and distribution of Mg atoms. As can be seen, Mg is mostly distributed in Al_x_Ga_1−x_N as intended.

### Hall measurement and two-carrier-species Hall-effect model

In many previous reports^4^,^13^,^14^,^15^, the standard Hall model was used to analyze the carrier concentration and mobility in superlattice-like structures. However, it should be noted that a simple Hall measurement gives no thickness information, therefore can only determine sheet hall concentration. As a result, it is difficult to delineate the contribution from bulk carriers and two-dimensional carrier gases.

**Figure 5:**
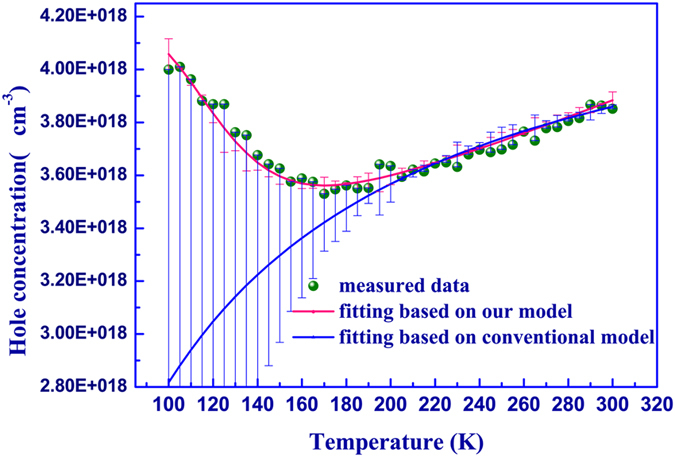
Ho le concentration as a function of temperature. The fitting curves are shown as solid lines using conventional hall-effect model and two-carrier-species Hall-effect model.

To address this issue, we quantitatively determine both the bulk and two-dimensional carrier properties by firstly applying a two-carrier-species (2D and bulk carriers) Hall-effect model. The measured hole concentrations are shown in Fig. 5, which exhibits a very weak dependence on temperature. However, if one closely looks at the hole concentration as a function of temperature, a more complicated behavior can be revealed. At relative high temperatures, 300 K to 200 K, a slight freeze-out effect is observed. However, on further decrease of the temperature, an abnormal increase of hole concentration (usually known as the freeze-in effect) is observed. Similar hole freeze-in behavior at low temperatures has also been observed in many previous reports^4^,^15^,^27^. Unfortunately, most earlier observations of this effect are not discussed in detail or are simply attributed to donor compensation. They argue that, as the thermally activated acceptors freeze out with decreasing temperature, compensating donors begin to have more effect on the conduction. However, based on previous reports even in n-type GaN without obvious compensating effects, such abnormal freeze-in behaviors can also be observed^27^. This phenomenon therefore deserves further attention. Furthermore, in our superlattice-like structures, besides bulk holes, parallel sheets of 2D hole gases can also be created at the interface of heterojunctions. It does not make sense to ignore the obvious differences in the electrical properties between them.

**Figure 6:**
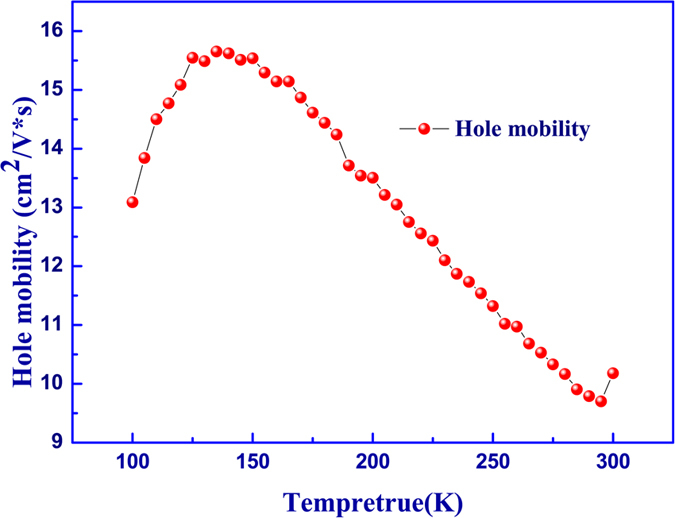
Hole mobility as a function of Temperature.

Here, two-carrier-species Hall-effect model is proposed to analyze the electrical behaviors of our Al_x_Ga_1−x_N/GaN sample. As shown in Fig. 5, the value of 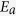 (acceptor ionization energy of bulk holes) and 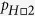 (sheet Hall concentration of 2D holes) can be obtained iteratively. It is observed that the conventional Hall-effect model is in agreement with experimental data at high temperatures (above 200 K), but decreasing temperature leads to a significant departure of the calculated concentration from that observed experimentally. Meanwhile, our model does agree with the measured experimental data very well at both low and high temperatures. The fitting parameters 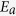 and 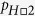 are about 60 meV and 8.36 × 10^13^ cm^−2^, respectively. Based on the single acceptor model, the bulk hole concentration is calculated to be about 1.14 × 10^18^ cm^−3^ at 300 K, which is about one order of magnitude higher than that of the normal p-type sample prepared by the same tools. The measured hole mobility in our sample is shown in Fig. 6. The relatively low hole mobility is similar to that reported by many others, which could be attributed to the high effective mass of holes in the minibands of the superlattice and/or alloy scattering^15^,^28^. The temperature dependence of mobility is much more complicated, which is related to several different scattering mechanisms and beyond the scope of this work. For simplicity, the measured hole motilities observed here can be understood as the average mobility of bulk holes and 2D hole gases.

## Discussion

Similar 2D carrier gases have been widely reported in many previous works in both p-type and n-type materials^13^,^27^, which can be attributed to polarization doping. In this work, we are more concerned with the abnormal high bulk hole concentration observed. As discussed above, we propose that this is the result of impurity resonant state p-type doping, which increases the overall bulk hole concentration by transforming the localized impurity states in barriers into resonant states in wells through orbital hybridization between Mg 2*p* and host N 2*p* orbitals. The underlying physical mechanism of this effect can also be understood in another way: in this new scenario, the deep acceptors in the barrier layers ionize into the valence band of the neighboring narrow band-gap material, rather than into its own, deeper, valance band. The high bulk hole concentration is strong evidence to support our theory that high efficiency p-type doping can be achieved by impurity resonant states in superlattice structures. Furthermore, we would like to point out that our approach can be considered as one special form of modulation doping. However, the purpose here is to generate high concentration carriers in wide band gap nitrides, which is otherwise difficult by directly doping the material itself, whereas the modulation doping is typically used to separate the dopant ions from the carriers in order to achieve high carrier mobility in the well, as wildly studied in arsenide or Ge/Si^29^,^30^.

In summary, a novel strategy for efficient p-type doping was proposed to overcome the fundamental problem of high activation energy in high bandgap III-nitrides by introducing impurity resonant states in an Mg doped Al_x_Ga_1−x_N/GaN superlattice structure. The characteristics and distribution of Mg impurity states were analyzed using first-principle calculations. Our results indicated that coupling and hybridization between Mg 2*p* impurity states and N 2*p* states is likely to be the main reason for the delocalized characteristics of the Mg impurity states. As a result, the wave-functions of Mg impurity states in the barrier layers are able to overlap with each other, then extended into well layers and act as resonant states. Therefore, a high hole concentration (about one order of magnitude higher than normal bulk Mg doped nitrides) could be successfully realized. This structure can be used to achieve efficient nitride based optoelectronic devices, especially in the deep ultraviolet wavelength range. The concept of impurity resonant state p-type doping presented here could also be applied to the production of highly p-type conductors in other wide-band-gap materials. The optimization on the thickness and components of Al_x_Ga_1−x_N/GaN structures is highly desired to obtain higher hole concentrations, as will be investigated in the subsequent works. Finally, the two-carrier-species Hall-effect model was used to extract the electrical parameters of bulk holes and 2D hole gases in superlattice-like structures, respectively. The model reported here can also be used to explain the abnormal and seldom analyzed freeze-in effect observed in many previous reports.

## Methods

### First-principles calculations

The characteristics of Mg impurity resonant states are studied using the first principles calculation, based on a density functional theory (DFT) encoded in the plane-wave based Vienna Ab initio Simulation Package (VASP)^31^. In these calculations, the generalized gradient approximations (GGA) of Perdew-Burke-Ernzerhof (PBE) functionals are used for the exchange correlation potential^32^. The cutoff energy is chosen to be 800 eV. For relaxed structures, the atomic forces are less than 0.03 eV/A. For simplicity, an AlN/GaN superlattice structure was examined using 2 × 2 × 10 supercell models rather than an Al_x_Ga_1−x_N/GaN structure.

### Two-carrier-species Hall-effect analysis

The relevant relationships for a two-carrier-species Hall-effect analysis can be expressed as^33^:


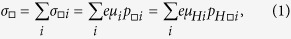



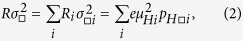


where: 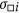, 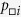, 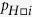 respectively represent the true sheet conductivity, the sheet concentration and the sheet Hall concentration of carrier *i*; and 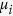, and 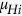 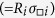 are respectively the mobility and Hall mobility of carrier *i*. In our model, *i* = 1 represents bulk holes, and *i* = 2 represents 2D holes. The Hall factor is close to unity as many other previous studies have used. We divide Eqs (1) and (2) by d, the thickness of Al_x_Ga_1−x_N/GaN structure to obtain the normally measured quantity, Hall concentration 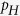 as:


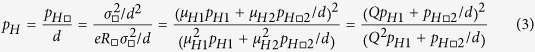


Here, *Q* is equal to 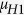/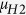, and is the ratio of bulk and 2D carrier Hall mobility.

The bulk hole concentration 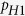 in a semiconductor with acceptor concentration N_A_ and acceptor ionization energy *E*_a_ can be expressed as^34^,^35^:


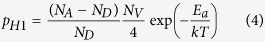


where 
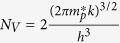
 is the effective density of states at the valance band edge of GaN; *m*_p_^*^ is the effective mass for holes ; g is the acceptor degeneracy (g = 4); T is the temperature; and h and k_B_ are Planck’s and Boltzmann’s constants respectively. Here, we assume that the concentration of 2D hole gases is temperature independent. Consequently, substituting Eq. (4) into Eq. (3) we obtain the numerical relationships between the Hall concentration 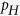 and T. From this expression, the value of 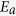 and 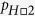 can be obtained iteratively.

## Additional Information

**How to cite this article**: Liu, Z. *et al.* Impurity Resonant States p-type Doping in Wide-Band-Gap Nitrides. *Sci. Rep.* **6**, 19537; doi: 10.1038/srep19537 (2016).

## Figures and Tables

**Figure 1:**
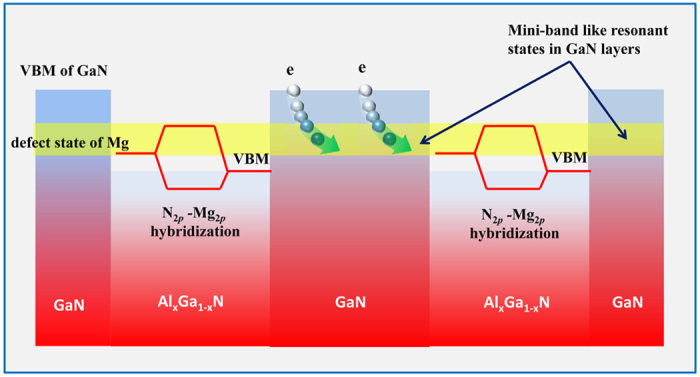
Schematic model showing the mechanism of impurity resonant states p-type doping. Schematic model showing the position and the hybridization between Mg p-like impurity states and valance band maximum of Al_x_Ga_1−x_N/GaN superlattice. Grey balls represent electrons and holes. Note that the initially localized impurity states in Al_x_Ga_1−x_N/GaN barrier layers transform into resonant states in GaN layers due to the hybrid orbitals. In this scenario, electrons will drop from the VBM of GaN into the impurity states or band without any energy barriers.
