# Corrigendum: Impurity Resonant States p-type Doping in Wide-Band-Gap Nitrides

**DOI:** 10.1038/srep23950

**Published:** 2016-04-20

**Authors:** Zhiqiang Liu, Xiaoyan Yi, Zhiguo Yu, Guodong Yuan, Yang Liu, Junxi Wang, Jinmin Li, Na Lu, Ian Ferguson, Yong Zhang

Scientific Reports 6: Article number: 1953710.1038/srep19537; published online: 01182016; updated: 04202016.

The original version of this Article contained typographical errors in the spelling of the author Guodong Yuan, which was incorrectly given as Gongdong Yuan. This has now been corrected in the PDF and HTML versions of the Article.

